# Long-Term Impact of Neonatal Intake of Oleanolic Acid on the Expression of AMP-Activated Protein Kinase, Adiponectin and Inflammatory Cytokines in Rats Fed with a High Fructose Diet

**DOI:** 10.3390/nu11020226

**Published:** 2019-01-22

**Authors:** Mashudu Given Matumba, Ademola Olabode Ayeleso, Trevor Nyakudya, Kennedy Erlwanger, Novel N. Chegou, Emmanuel Mukwevho

**Affiliations:** 1 Department of Biochemistry, Faculty of Natural and Agricultural Science, North West University, Mafikeng Campus, Private Bag X2046, Mmabatho 2735, South Africa; mgmatumba@gmail.com; 2 Department of Biochemistry, Faculty of Science, Adeleke University, P.M.B. 250, Ede, Osun State 232101, Nigeria; ademola.ayeleso@adelekeuniversity.edu.ng; 3 Department of Human Anatomy and Physiology, Faculty of Health Sciences, University of Johannesburg, Doornfontein, Johannesburg 2028, South Africa; trevorn@uj.ac.za; 4 School of Physiology, Faculty of Health Sciences, University of the Witwatersrand, Parktown, Johannesburg 2193, South Africa; kennedy.erlwanger@wits.ac.za; 5 DST-NRF Centre of Excellence for Biomedical Tuberculosis Research and South African Medical Research Council Centre for Tuberculosis Research, Department of Biomedical Sciences, Division of Molecular Biology and Human Genetics, Faculty of Medicine and Health Sciences, Stellenbosch University, Tygerberg 7505, South Africa; novel@sun.ac.za

**Keywords:** type-2 diabetes, obesity, oleanolic acid, metabolic syndrome, high fructose diet, AMPK, adiponectin, inflammatory cytokines

## Abstract

AMP-activated protein kinase (AMPK) is known to regulate both glucose and lipid metabolism, which play vital roles in the development of metabolic syndrome. One way of regulating AMPK is through hormonal activation using adiponectin. Patients diagnosed with type-2 diabetes (T2D) and obesity exhibit low adiponectin concentration levels in their blood. Moreover, studies have also shown that inflammatory processes play a significant role in the etiology of these metabolic diseases. In this study, the long-term effects of neonatal intake of oleanolic acid (OA) on the *AMPK* gene, genes associated with glucose transport and lipid metabolism, adiponectin levels, and inflammatory biomarkers in rats fed with a high fructose diet were investigated. Seven day old pups were randomly divided into five groups and treated as follows; 0.5% dimethylsulphoxide *v*/*v* in distilled water vehicle control (CON), oleanolic acid (OA, 60 mg/kg), high fructose diet (HF, 20% *w*/*v*), high fructose diet combined with oleanolic acid (HF+OA), and high fructose diet combined with metformin (HF+MET, 500 mg/kg). The treatments were administered once daily until day 14. The rats were then weaned at day 21 and fed standard rat chow and had *ad libitum* access to plain drinking water until day 112. The quantitative polymerase chain reaction (qPCR) was used to analyze the gene expressions of *AMPK*, *Glut-4*, *Cpt-1*, *AdipoR1*, *AdipoR2*, *TNF-α*, and *IL-6* in the skeletal muscles. Bio-Plex Pro magnetic bead-based assay was used to measure plasma levels of inflammatory markers (TNF-α, IL-6, VEGF, and MCP-1) while ELISA kits were used to measure adiponectin concentration in blood plasma. The results obtained in this study showed that neonatal supplementation with OA significantly increased *AMPK* gene expression approximately ~4-fold in OA fed rats compared to those that were fed with HF alone. In addition, glut-4 gene expression was also significantly higher in the OA treatment group compared to all the other experimental groups except the CON group whereas *Cpt-1* gene was more expressed when OA was administered alone. Together, these results indicated that OA can play a role in glucose and lipid metabolism gene regulation. Furthermore, the results showed that the OA group had ~1.5-fold increase in adiponectin concentration when comparedto the HF group. Moreover, HF increased levels of inflammatory cytokines, which was attenuated by neonatal administration of OA. Plasma concentration and gene expression in the skeletal muscle for TNF-α and IL-6 were significantly increased in rats that were treated with HF alone when compared to all the other groups. On the contrary, the high levels of TNF-α and IL-6 were reduced when OA was administered. These findings suggest that intake of oleanolic acid during the neonatal stage of development could be a potential strategic intervention for the long-term prevention of metabolic diseases such as T2D and obesity.

## 1. Introduction

Type 2 diabetes (T2D) is an epidemic health challenge all over the world as a result of the high prevalence of obesity [1]. It is important to note that obesity and T2D are causally linked through their association with the development of skeletal muscle insulin resistance [2]. Insulin resistance is described as a condition in which circulating insulin becomes non-functional at target tissues, namely skeletal muscle, adipose tissue, and the liver [3]. T2D was initially thought to be a disease exclusive to adults, however there is a rising incidence of T2D in adolescents in parallel with the increasing prevalence of obesity amongst children [4]. Consequently, there is huge interest in studies within this field to elucidate mechanisms of this epidemic [5].

AMP-activated protein kinase (AMPK) is a well-studied and described enzyme which serves asthe master regulator of both lipid and glucose metabolic pathways. An extensive literaturesurvey has shown that dysregulation of AMPK plays a critical role in the etiology of T2D and obesity [6]. Once AMPK is activated, it increases both fatty acid uptake and β-oxidation in the mitochondria with a concomitant increase in glucose uptake through the translocation of glucose transporter 4 (GLUT-4). Glut-4 is the major glucose transporter protein found in the intercellular vesicles across the plasma membrane in the skeletal muscles [7]. In T2D, translocation of Glut-4 is limited, resulting in hyperglycaemia. Excess lipids also play a significant role in the development of T2D. Carnitine palmitoyltransferase (CPT-1) is the gatekeeper for the entry of fatty acids into the mitochondria [8] for oxidation. Both lipid oxidation and glucose uptake are impaired in T2D and obese persons, owing to the fact that these two pathways are regulated together by AMPK. As such, this enzyme has now become a vital target in the development of therapeutics much needed to ameliorate the health outcomes associated with T2D and obesity [6].

The adipose tissue secretes various bioactive proteins and hormones into the circulation named adipo-cytokines essential in T2D [9]. One such critical hormone secreted by the adipose tissue is adiponectin. Adiponectin is a protein hormone that plays an important role in the modulation of glucose and lipid metabolism in insulin-sensitive tissues in both humans and animals [9,10]. Furthermore, literature has reported that adiponectin is associated with obesity-related insulin resistance and T2D [11]. It has been demonstrated that adiponectin’s mechanism of action is through activation of AMPK, which then regulates both glucose and lipid metabolism [12]. Lastly, research studies have also shown that patients suffering from T2D and obesity related to insulin resistance are associated with decreased adiponectin concentrations in circulating blood [13]. The reason for this is that obesity and T2D can result from adiponectin deficiency, thus making this hormone a good target for possible therapeutic interventions, focusing on the possibility that adiponectin treatment may improve T2D and obesity-related insulin resistance [11].

Inflammatory processes also play a significant role in the etiology of T2D and obesity [14]. Consequentially, T2D is associated with an increased circulating concentration of inflammatory biomarkers [15]. For example, tumor necrosis factor-α (TNF-α) is an adipo-cytokine involved in inflammation and is found to be increased in the metabolic syndrome, T2D and obesity, and therefore contributes to insulin resistance by interfering with insulin receptor signaling [16,17]. Furthermore, it has also been shown that IL-6 is elevated in insulin resistant and obese individuals and as such, its plasma levels correlate with the degree of insulin resistance [6]. In this present study, we also analyzed the blood plasma concentration of monocyte chemoattractant protein-1 (MCP-1) and vascular endothelial growth factor (VEGF). The monocyte chemoattractant protein-1 (MCP-1), which signals to macrophages through the C-C chemokine receptor 2 (CCR2), is strongly correlated with T2D and obesity [18]. Circulating MCP-1 has been found significantly increased in patients with T2D [19]. Similarly, another study showed that plasma VEGF concentration was high in diabetic patients who were hospitalized because of poor glycaemic control compared to the concentration in healthy subjects [20]. Vascular endothelial growth factor (VEGF) is one of the major growth factors that have an adverse effect on ocular neovascular diseases such as diabetic retinopathy [21].

Current therapeutic approaches in the management of metabolic dysfunction involve the use of conventional drugs. The use of these pharmaceutical drugs has been associated with the development of undesirable adverse side-effects [22]. Moreover, their cost is beyond the reach of many, particularly in resource-limited African rural and some urban areas. As such, most rural and urban based people make use of plant based ethno-medicines for their primary health care. The efficacy of ethno-medicines in the management of several metabolic disorders has been attributed to the presence of bioactive phytochemicals [22]. One such phytochemical, that we selected for the current study, is oleanolic acid (OA).

Oleanolic acid (OA) is a bioactive pentacyclin triperpenoid compound isolated from plants belonging to the family *Oleaceae*. OA is known to exert anti-diabetic effects among other health promoting characteristics, with no adverse effects [23,24]. Oleanolic acid has also been shown to significantly lower blood glucose and weight loss in diabetic rats [25]. However, its actual mechanism of action in conferring the anti-diabetic effects is not well defined. Most studies that investigated the therapeutic efficacy of OA made use of adult animal experimental models. Until recently in studies by references [26,27], no studies had been conducted targeting the neonatal period, a critical window of developmental plasticity. Therefore, the aim of this present study was to investigate the long-term impact of neonatal intake of OA on the development of metabolic diseases such as T2D and obesity in rats that were fed a high fructose diet.

## 2. Materials and Methods

### 2.1. Animals

This study was conducted using forty male Sprague Dawley rat pups obtained from the Central Animal Services of the University of the Witwatersrand, South Africa. Seven day old pups were randomly divided into the five groups namely the 0.5% *v*/*v* dimethylsulphoxide in distilled water vehicle control (CON), Oleanolic acid (OA, 60 mg/kg), High fructose diet (HF, 20% *w*/*v*), High fructose diet combined with oleanolic acid (HF+OA), and High fructose diet combined with metformin (HF+MET, 500 mg/kg). The doses of oleanolic acid and metformin used werepreviouslyused in other metabolic studies in rats [28,29]. The treatments were administered by orogastric gavage (10 mL/kg) once dailyuntil day 14. This initial treatment targeted one of the windows of developmental plasticity during which interventions can induce epigenetic changes that have long lasting outcomes on the disease state or health of the individual [30]. The rats were then weaned on day 21 and fed with standard rat chow and had *ad libitum* access to plain drinking water until day 111 to investigate whether the neonatal interventions could have an impact on health outcomes later in life. At the end of the experiment on day 112, the rats were euthanized with sodium pentobarbital (200 mg/kg body weight; Euthanaze^®^, Centaur Laboratories, Johannesburg, South Africa). Blood was collected by cardiac puncture and samples were dispensed into plain and heparin coated tubes. The blood samples were centrifuged and the plasma and serum then collected. Skeletal muscle samples were dissected out, snap frozen in liquid nitrogen, and stored at −80 °C until further assays were performed. All animal experiments were conducted in accordance with protocols approved by theAnimal Ethics Screening Committee (AESC) of the University of Witwatersrand, Johannesburg, South Africa (AESC approval number 2014/47/D).

### 2.2. Measurement of Adiponectin Concentration Using ELISA

A rat adiponectin ELISA kit (Sigma-Aldrich, St. Louis, MO, USA) was used to measure adiponectin concentration in blood plasma according to the manufacturer’s protocol. Briefly, 1× wash buffer was prepared by diluting the 20× wash buffer with deionized water and the 1× ELISA buffer was also prepared by diluting the 5× ELISA buffer with deionized water. The rat adiponectin protein standard was reconstituted with 1 mL of deionized water and serial dilutions of the standard were prepared. Then 1× biotinylated rat adiponectin detection antibody solution was prepared by diluting the detection antibody 1:1000 in 1× ELISA buffer and 1× HRP-Streptavidin solution was prepared by diluting HRP solution 1:100 in 1× ELISA buffer.

The protein standard and samples were added into appropriate wells (100 µL to each well) and the plate was incubated at room temperature for 2.5 h with gentle shaking. The solution was discarded and the wells were washed 4 times with 300 µL 1× wash solution. Then 100 µL of 1× prepared biotinylated detection antibody was added to each well and the plate was incubated for 1 h at room temperature with gentle shaking. The solution was discarded and the wells were washed 4 times with 300 µL 1× wash solution again. Then 100 µL of the prepared HRP-Streptavidin solution was added to each well and the plate was incubated for 45 min at room temperature with gentle shaking. The solution was discarded and the wells were washed 4 times with 300 µL 1× wash solution for the last time. Then 100 µL of ELISA colorimetric TMB reagent was added to each well and the plate was incubated for 30 min at room temperature in a dark room with gentle shaking. Lastly, 50 µL of stop solution was added to each well and absorbance was measured at 450 nm immediately with a spectrophotometer. The protein standard curve was generated and the unknown concentration of the samples was determined.

### 2.3. Measurement of Gene Expression Using qPCR Analysis

#### 2.3.1. RNA Extraction

Total RNA was isolated using Trizol (Life Technologies, Carlsbad, CA, USA). The frozen skeletal muscle tissue was crushed in liquid nitrogen using a pestle and mortar and, 1 mL of trizol was added to 200 mg of tissue sample. The mixture was homogenized for 3 min while working on ice. The homogenate was then vortexed vigorously after addition of 200 µL of chloroform and then incubated on ice for 15 min. The homogenate was then centrifuged at 12,000× *g* for 15 min at 4 °C to get the phase spectrum. The aqueous phase was then transferred to a new fresh tube. The total RNA was precipitated by incubating the solution for 10 min in ice after addition of 0.5 mLof isopropanol. After incubation, the solution was centrifuged for 10 min at 12,000× *g* at 4 °C and the supernatant was removed. The pellet was washed with 70% ethanol and then centrifuged for 10 min at 7500× *g* at 4 °C. The supernatant was removed and the total RNA pellet was dissolved in 50 µL of RNase free water.

Total RNA concentration and quality was measured using the NanoDrop Lite Spectrophotometer (Thermo Scientific, Johannesburg, South Africa). The integrity of the total RNA was analyzed using 1% agarose gel electrophoresis; briefly 1 g of agarose was dissolved in 100 mLof TAE buffer by heating the mixture with a microwave for 60 s. The mixture was allowed to cool down to at least 50 °C, and then 1.5 µL of ethidium bromide was added. The solution was poured in the gel tray, the comb was inserted, and the gel was allowed to solidify. The samples were loaded in the wells after being mixed with the loading dye. The electrophoresis was run for 45 min at 100 volts and TAE buffer was also used as a tank buffer. The gel was viewed under the doc machine (Bio-Rad Laboratories, Hercules, CA, USA). 

#### 2.3.2. cDNA Synthesis

The cDNA was synthesized using the SuperScript VILO cDNA Synthesis kit (Thermofisher Scientific, Johannesburg, South Africa) according to the manufacturer’s protocol. Briefly, master mix was prepared by mixing together the 5× VILO Reaction mix and the 10× SuperScript enzyme mix while working on ice. The volume of RNA required to make a concentration of 2.5 µg was calculated and DEPC-treated water was used to compensate the volume to a final of 20µL in all reaction tubes. The tube contents were gently mixed and then incubated for 10 min at 25 °C, then followed by 42 °C incubation for 60 min. The reaction was terminated by incubation at 85 °C for 5 min. All incubations were done in the T100 Thermal Cycler (Bio-Rad, Singapore). The cDNA was stored at −20 °C until use.

#### 2.3.3. Real-Time PCR

Real-Time PCR was performed using the PowerUp SYBR Green master mix (Applied Biosystems, Life Technologies, USA) according to the manufacturer’s protocol. The primers (Table 1) and the PowerUp SYBR Green master mix were mixed together appropriately according to the volumes given by the manufacturer. The cDNA template was mixed with RNase-free water. Solutions were then transferred to appropriate wells in the 96 well plate and the template was added lastly. The plate was then sealed with an optical adhesive cover. The step One plus Real-Time PCR system thermal cycling block instrument (Applied Biosystems, Life Technologies, USA) was used. The reaction plate was placed in the instrument and the thermal cycling conditions were set then the run was started.

### 2.4. Measurements of Inflammatory Markers Concentration in Blood Plasma

Bio-Plex Pro magnetic bead-based assays (Bio-Rad Laboratories, Hercules, CA, USA) were used to measure plasma levels of inflammatory markers (TNF-α, IL-6, VEGF, and MCP-1) using the Luminex technology. Samples were evaluated undiluted. Briefly, samples were added to a mixture of fluorescent magnetic beads bound with specific anticytokine primary antibodies, resulting in binding of the cytokines to the bead with the corresponding antibody. The biotinylated anticytokine secondary antibodies were then added and allowed to bind to the cytokine-bead complex. After washing, followed by the addition of fluorescent phycoerythrin-conjugated streptavidin, assays were read on the Bio Plex 200 platform (Bio Rad Laboratories, Hercules, CA, USA). The standard curve for all the analytes ranged from 3 to 12,000 pg/mL. Bio-Plex manager software version 6.1 (Bio Rad Laboratories, USA) was used for bead acquisition and analysis.

### 2.5. Statistics

Data were expressed as mean ± SD. Analysis of statistical significance of differences in measurements between samples was done by one-way ANOVA (GraphPad Prism version 6). *p*<0.05 was considered statistically significant.

## 3. Results

### 3.1. Gene Expression of AMPK

**Figure 1:**
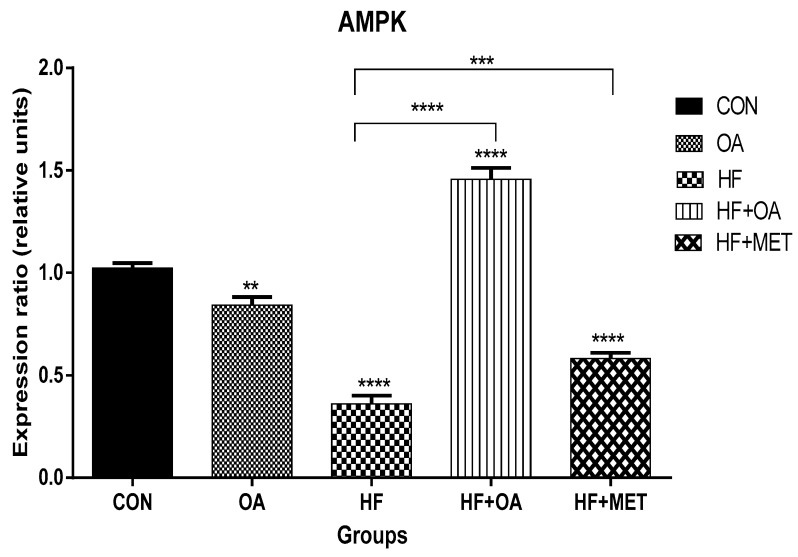
Gene expression of *AMPK* gene in the skeletal muscles of rats. The expression ratio of the gene is presented in relative units. Data is presented as mean ± SD. The *p* value representing the degree of significance difference amongst the groups is indicated as ** *p* < 0.01; *** *p* < 0.001; **** *p* < 0.0001. CON (Control), OA (Oleanolic acid), HF (High fructose diet), MET (Metformin).

Figure 1 shows the influence of OA on *AMPK* gene expression in HF-induced rats. The results in Figure 1 show the gene expression of *AMPK* in the skeletal muscles. Neonatal administration of fructose (HF) significantly decreased *AMPK* gene expression when compared to the control group. However, neonatal supplementation with oleanolic acid (HF+OA) resulted in ~4-fold increase of *AMPK* gene expression when compared to the HF group. Furthermore, rats given metformin and the high fructose diet (HF+MET) showed ~1.6-fold increase of *AMPK* gene expression when compared to the HF group.

### 3.2. Gene Expression of Glut-4 and Cpt-1

**Figure 2:**
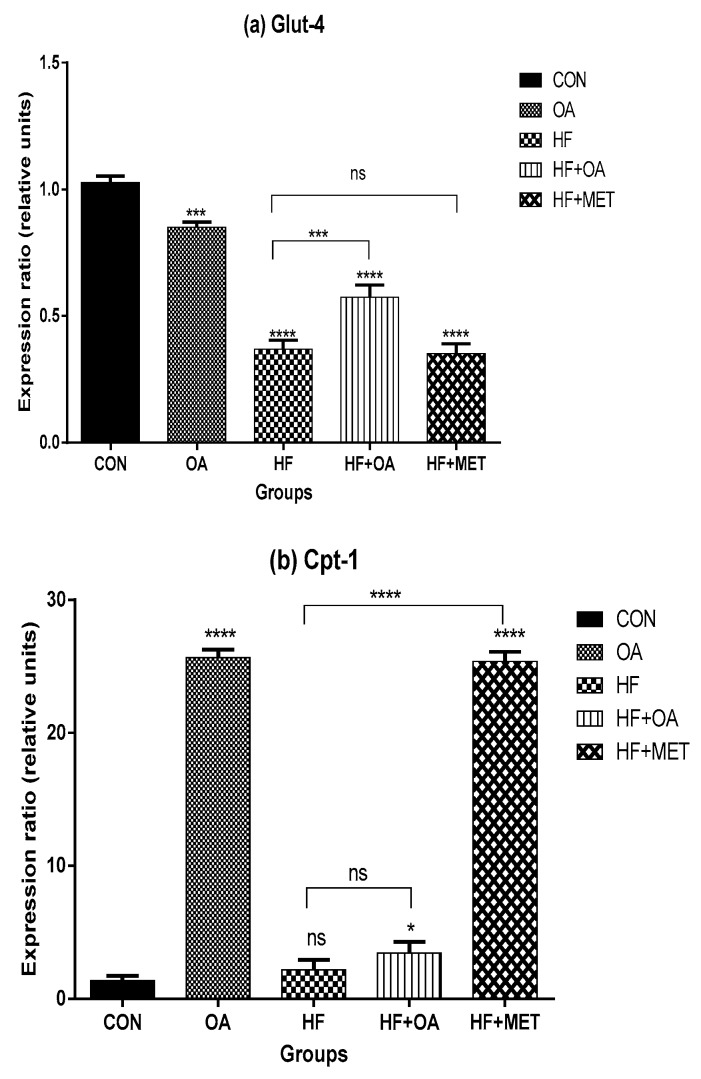
Gene expression of *Glut-4* (**a**) and *Cpt-1* in the skeletal muscles (**b**). The expression ratio of the gene is presented in relative units. Data is presented as mean ± SD. The *p* value representing the degree of significance difference amongst the groups is indicated as * *p* < 0.1; *** *p* < 0.001; **** *p* < 0.0001; ns = no significant difference. CON (Control), OA (Oleanolic acid), HF (High fructose diet), MET (Metformin).

Figure 2a shows the *Glut-4* gene expression while Figure 2b shows the *Cpt-1* gene expression in the skeletal muscles. *Glut-4* gene expression was significantly higher in the OA treatment group compared to all the other experimental groups, except for the CON group. The HF+OA group showed ~1.5-fold *Glut-4* gene expression when compared to the HF group whereas there was no significant difference in the *Glut-4* gene expression between the HF and HF+MET group (Figure 2a).

The rats that were treated with OA neonatally showed a markedly significant ~18.8-fold increase in *Cpt-1* gene expression when compared to the CON group whereas the HF+MET group had ~18.5-fold increase in *Cpt-1*gene expression when compared to the CON group. The HF group had the lowest *Cpt-1*gene expression when compared to all the other groups except with the CON group. Both oleanolic acid and metformin were able to enhance the *Cpt-1*gene expression (Figure 2b).

### 3.3. Adiponectin Concentration

**Figure 3:**
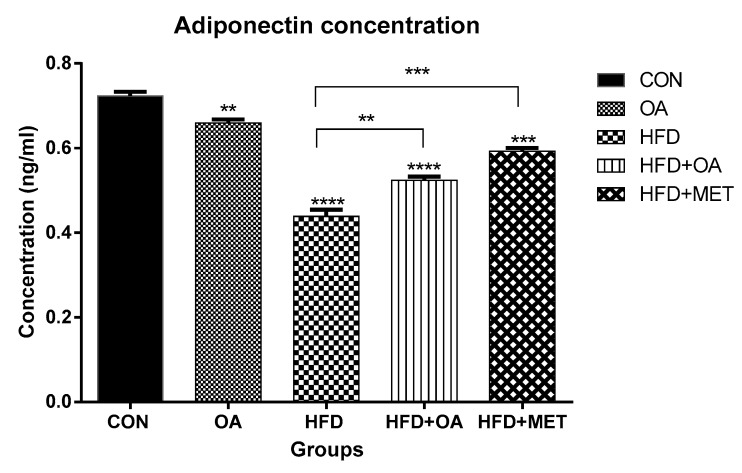
Concentration of adiponectin in the blood plasma. Concentration is presented in ng/Ml and data is presented as mean ± SD. The *p* value representing the degree of significance difference amongst the groups is indicated as ** *p* < 0.01; *** *p* < 0.001; **** *p* < 0.0001. CON (Control), OA (Oleanolic acid), HF (High fructose diet), MET (Metformin).

Figure 3 shows the concentration of adiponectin in blood plasma in response to various treatments. In this study, neonatal treatment with HF significantly decreased the concentration of plasma adiponectin compared to all the other groups (*p* < 0.05). The OA group had ~1.5-fold increase in adiponectin concentration when compared to the HF group. Neonatal treatment with oleanolic acid (HF+OA) and metformin (HF+MET) prevented the fructose-induced decrease in adiponectin levels and caused ~1.5-fold and ~1-fold increase respectively, in adiponectin concentration.

### 3.4. Gene Expression of Adiponectin Receptors

**Figure 4:**
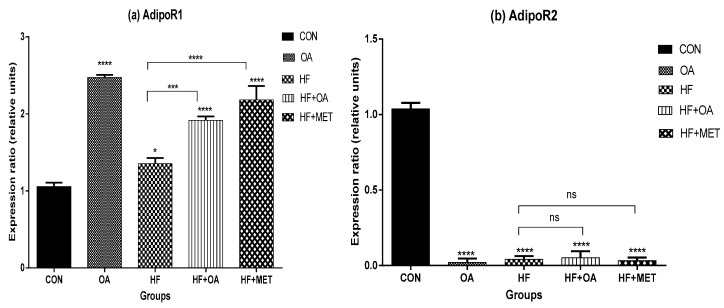
The gene expression of *AdipoR1* (**a**) and *AdipoR2* in the skeletal muscles (**b**). The expression ratio of the gene is represented in relative units. Data is represented as mean ± SD. The *p* value representing the degree of significance difference amongst the groups is indicated as * *p* < 0.1; *** *p* < 0.001; **** *p* < 0.0001; ns = no significant difference. CON (Control), OA (Oleanolic acid), HF (High fructose diet), MET (Metformin).

Figure 4a shows the *AdipoR1* gene expression while Figure 4b shows the *AdipoR2* gene expression in the skeletal muscles. High fructose diet significantly decreased the *AdipoR1* gene expression when compared to all the other treatment groups except for the CON group. The OA group showed ~2-fold increase in *AdipoR1* gene expression when compared to the CON group. In addition, the HF+OA and HF+MET groups showed ~1.4 and ~1.6-fold increases respectively in *AdipoR1* gene expression when compared to the HF group (*p* < 0.05). These results indicate that *AdipoR1* gene was more expressed following neonatal treatments with OA and MET, whereas HF negatively decreased its expression as shown in Figure 4a. However, *AdipoR2* was not well expressed in all the groups compared to the CON group. Furthermore, there was no significance difference in *AdipoR2* gene expression on rats fed with high fructose diet (HF) when compared to rats in groups that received treatment (HF+OA and HF+MET) (Figure 4b).

### 3.5. Inflammatory Biomarkers Concentration and Gene Expression

**Figure 5:**
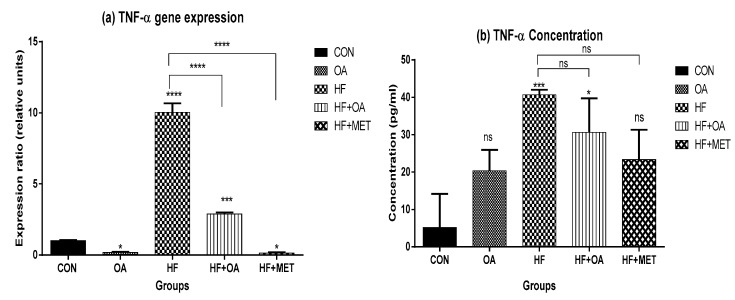
Gene expression of *TNF-α* in the skeletal muscle (**a**) Concentration of TNF-α in the blood plasma (**b**). The expression ratio of the gene is presented in relative units while concentration is in pg/mL and data is presented as mean ± SD. The *p* value representing the degree of significance difference amongst the groups is indicated as * *p* < 0.1; *** *p* < 0.001; **** *p* < 0.0001; ns = no significant difference. CON (Control), OA (Oleanolic acid), HF (High fructose diet), MET (Metformin).

Figure 5a shows the skeletal muscle *TNF-α* gene expression and Figure 5b shows the concentration of TNF-α in the blood plasma. *TNF-α* gene expression was significantly increased in rats that were treated with HF alone when compared to all other groups. Neonatal treatment with OA and MET prevented the fructose-induced increase in the expression of *TNF-α*. The HF group had ~7.8-fold increase in TNF-α concentration when compared to the CON group. On the other hand, rats treated with OA only had the lowest TNF-α concentration when compared to other treatment groups. Furthermore, HF+MET and HF+OA group had lower TNF-α concentration when compared to the HF group.

**Figure 6:**
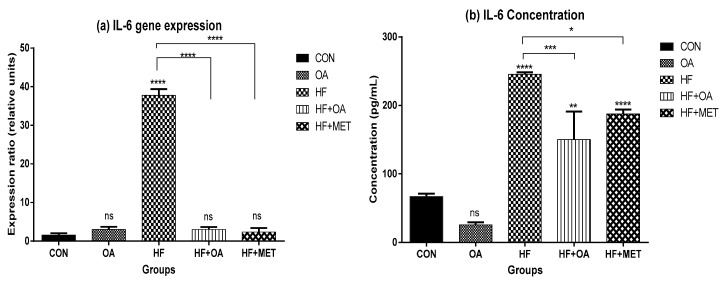
(**a**) Gene expression of *IL-6* in the skeletal muscles; (**b**) Concentration of IL-6 in blood plasma. The expression ratio of the gene is presented in relative units while concentration is presented in pg/mLand data is represented as mean ± SD. The *p* value representing the degree of significance difference amongst the groups is indicated as * *p* < 0.1; ** *p* < 0.01; *** *p* < 0.001; **** *p* < 0.0001; ns = no significant difference. CON (Control), OA (Oleanolic acid), HF (High fructose diet), MET (Metformin).

In Figure 6a, there was ~24-fold increase of *IL-6* gene expression in the skeletal muscles of fructose-fed rats when compared to all other groups. Neonatal treatment with oleanolic acid (HF+OA) and metformin (HF+MET) reversed the high expression of *IL-6* expression induced by high fructose diet (HF). Figure 6b shows that the HF treated rats had ~3.7-fold IL-6 concentration increase when compared to the CON group. Both HF+OA and HF+MET groups had low IL-6 concentrations when compared to the HF group.

**Figure 7:**
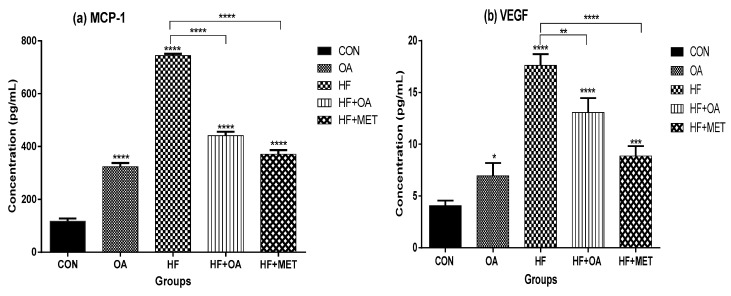
Concentration of MCP-1 (**a**) and VEGF in the blood plasma (**b**). Concentration is presented in pg/mLand data is presented as mean ± SD. The *p* value representing the degree of significance difference amongst the groups is indicated as * *p* < 0.1; ** *p* < 0.01; *** *p* < 0.001; **** *p* < 0.0001. CON (Control), OA (Oleanolic acid), HF (High fructose diet), MET (Metformin).

Figure 7a shows the concentration of IL-6 while Figure 7b shows the concentration of MCP-1 in the blood plasma. The results showed that neonatal fructose (HF) treatment caused a significant increase (~6.4-fold) in the concentration of MCP-1 when compared to the CON group (*p* < 0.05). The neonatal treatment with OA alone and a combination of high fructose diet with either oleanolic acid (HF+OA) or metformin (HF+MET) lowered MCP-1 concentration when compared to the CON and HF group. Figure 7b shows that fructose treatment (HF) caused a ~4.3-fold increase in the concentration of VEGF when compared to the control. Neonatal treatment with oleanolic acid alone (OA) and a combination of fructose with oleanolic acid (HF+OA) and metformin (HF+MET) lowered the VEGF concentration when compared to the HF.

## 4. Discussion

This study investigated the long-term impact of neonatal intake of oleanolic acid and fructose on the gene expression of *AMPK* and other genes that play a key role in glucose and lipid metabolism. Our findings show that oleanolic acid significantly increased *AMPK* gene expression with ~4-fold in high fructose-fed rats that were treated with oleanolic acid when compared to those that were fed with high fructose diet alone. The result reveals that oleanolic acid regulates AMPK and could be a potential therapeutic agent in the management of T2D and obesity. AMPK is a fuel sensor for glucose and lipid metabolism [31]. Activated AMPK in skeletal muscles stimulates glucose uptake by increasing GLUT-4 translocation and fatty acid oxidation in lipid metabolism [6]. In this study, oleanolic acid significantly increased *Glut-4* gene expression with ~1.5-fold in concomitantly high fructose-fed rats when compared to the rats that were fed with high fructose diet alone. Since GLUT-4 is seen as one of the targets for therapeutic modalities and was significantly increased by OA, these findings are a significant step towards the better management and treatment of T2D and obesity [32].

CPT-1 plays a crucial role in fatty acid β-oxidation as a gatekeeper for entry of fatty acids into the mitochondria [7]. This study showed no significant difference in *Cpt-1* gene expression between high fructose-fed rats that were treated with oleanolic acid and those that were fed with a high fructose diet alone. However, oleanolic acid administered alone was able to up-regulate *Cpt-1* gene expression, which promotes fatty acid uptake in the mitochondria for β-oxidation.

Findings from this study showed thatfructose reduced the concentration of adiponectin in plasma and downregulated the gene expression of its receptors (*AdipoR1* and *AdipoR2*) in skeletal muscles. Previous studies have shown that the concentration of adiponectin and its receptors are down-regulated in T2D and obesity-related insulin resistance [12,33]. Adiponectin has also been reported to improve insulin sensitivity and reduce the risk of developing T2D [34]. The present study showed that OA up-regulated adiponectin concentration by ~1-fold in rats that were fed a high fructose diet. Furthermore, for adiponectin to elicit its effects, it has to bind first to its receptors (AdipoR1 and AdipoR2). It was observed in this study that oleanolic acid significantly increased *AdipoR1* gene expression by ~1.4 fold in rats that were treated with oleanolic acid when compared to the rats that were given the high fructose diet alone. The improvement of *AdipoR1* gene expression in the skeletal muscle is a positive effect since adiponectin exerts its response through binding to *AdipoR1*. Therefore, the results shown in Figure 4a indicated that OA influenced the expression of *AdipoR1* gene expression in a positive manner. On the other hand, *AdipoR2* was poorly expressed in all the groups compared to the control. These findings confirm previous reports that showed that *AdipoR1* dominates in skeletal muscle whilst *AdipoR2* predominates in the liver tissue [17,35], thus the low expression of *AdipoR2* in skeletal muscle was not unexpected. Systemic inflammatory markers are risk factors for the development of T2D and metabolic dysfunction [36]. In this study, inflammatory cytokines were increased in rats that were fed with a high fructose diet. These results are consistent with other previous studies showing that inflammatory cytokine markers are elevated in insulin resistance and obese individuals [6,15,16,19,37]. High fructose diet induced elevation in cytokine levels and was attenuated when these rats were treated with oleanolic acid, indicating the potential of OA in ameliorating symptoms of T2D and obesity. High fructose effects are consistent with the notion that diet and in particular high carbohydrate levels, have deleterious effects on normal functioning of metabolic processes of the cell and are a major contributor to health problems such as T2D and obesity [38,39,40,41,42,43,44,45,46].

## 5. Conclusions

This study has shown that neonatal treatment with OA prevented the fructose-induced down regulation of *AMPK* expression in skeletal muscles and decreased plasma level of adiponectin in Sprague Dawley rats. Neonatal OA oral administration also prevented the fructose-induced increase in the level of inflammatory cytokines, and increased the expression glucose transporter 4 gene (*Glut-4*) that is responsible for glucose uptake and prevention of hyperglycaemia. These findings seem to suggest that oleanolic acid could be used as an alternative prophylactic therapeutic agent against the development of metabolic diseases such as T2D and obesity.

## Figures and Tables

**Table 1:** List of primers used in the study.

Genes	Forward Primer (F) and Reverse Prime (R)
*AdipoR1*	F: 3′-AAGCACCGGCAGACAAGAGC-5′ R: 3′-AGGAAGAACCAGCCCATCTG-5′
*AdipoR2*	F: 3′-CTGTGTGCTGGGCATTGCAG-5′ R: 3′-AGCCTATCTGCCCTATGGTG-5′
*AMPK-α*	F: 5′-GGCAAAGTGAAGATTGGAGAACA-3′ R: 5′-AACTGCCACTTTATGGCCTGT C-3′
*Glut-4*	F: 5′-GCAGCGAGTGACTGGACCA-3′ R: 5′-CCAGCCACGTTGCATTGTAG-3′
*CPT-1*	F: 5′-CGGTTCAAGAATGGCATCATC-3′ R: 5′-TCACACCCACCACCACGAT-3′
*TNF-α*	F: 5′-CATCTTCTCAAAATTCGAGTGACAA-3′ R: 5′-TGGGAGTAGACAAGGTACAACCC-3′
*IL-6*	F: 5′-GCCACTGCCTTCCCTACTTCA-3′ R: 5′-GACAGTGCATCATCGCTGTTCA-3′
*Actin*	F: 5′-GACGAGGCCCAGAGCAAGAGA-3′ R: 5′-GGGTGTTGAAGGTCTCAAACA-3′
